# Effectiveness of PCSK9 inhibitors versus statins in type 2 diabetes and dyslipidemia: a propensity-matched study

**DOI:** 10.3389/fendo.2025.1709009

**Published:** 2025-11-19

**Authors:** Jheng-Yan Wu, Yu Min Lin, Wan-Hsuan Hsu, Tinghui Liu, Ya-Wen Tsai, Po-Yu Huang, Min Hsiang Chuang, Tsung Yu, Chih-Cheng Lai

**Affiliations:** 1 Department of Nutrition, Chi Mei Medical Center, Tainan, Taiwan; 2 Department of Public Health, College of Medicine, National Cheng Kung University, Tainan, Taiwan; 3 Division of Cardiology, Department of Internal Medicine, Chi Mei Medical Center, Chiali, Tainan, Taiwan; 4 Department of Internal Medicine, Chi Mei Medical Center, Tainan, Taiwan; 5 Department of Psychiatry, Chi Mei Medical Center, Tainan, Taiwan; 6 Division of Preventive Medicine, Chi Mei Medical Center, Tainan, Taiwan; 7 Department of Intensive Care Medicine, Chi Mei Medical Center, Tainan, Taiwan; 8 School of Medicine, College of Medicine, National Sun Yat-sen University, Kaohsiung, Taiwan

**Keywords:** PCSK9 inhibitor, type 2 diabetes, All-cause mortality, major adverse cardiovascular event, major adverse kidney event

## Abstract

**Background:**

Proprotein convertase subtilisin/kexin type 9 inhibitors (PCSK9is) exhibit promising lipid-lowering activity, but evidence regarding their effectiveness in real-world diabetic populations is limited.

**Methods:**

Based on TriNetX database, individuals T2D and dyslipidemia who were newly prescribed either a PCSK9 inhibitor or a statin between January 1, 2015, and April 30, 2025, were identified. After propensity score matching, 20,489 patients were classified into each treatment group. Primary endpoints were defined as a composite of all-cause mortality, major adverse cardiovascular events (MACE), and major adverse kidney events (MAKE) during the 5-year follow-up.

**Results:**

PCSK9i use was associated with a reduced incidence of the primary outcome (hazard ratio [HR], 0.75; 95% CI, 0.70–0.81). Secondary outcomes also favored PCSK9i use, with reduced incidence of all-cause mortality (HR, 0.65; 95% CI, 0.60–0.705), MACE (HR, 0.83; 95% CI, 0.76–0.90), and MAKE (HR, 0.70; 95% CI, 0.61–0.81). Similar trends were observed for most of the subgroup and sensitivity tests. The association was significant for alirocumab and evolocumab, but not for inclisiran, likely due to limited sample size.

**Conclusions:**

Among patients with T2D and dyslipidemia, PCSK9i use was associated with reduced incidences of cardiovascular and renal events and all-cause mortality compared to statin therapy. These findings support the promising role of PCSK9is in high-risk diabetic populations.

## Introduction

Diabetes mellitus (DM) remains a public health challenge, as well as its prevalence and associated burden increasing steadily across the world. In 2021, approximately 529 million individuals were living with DM, and estimates suggest the number could surpass 1.3 billion by 2050 (1). The prevalence is particularly high in North Africa and the Middle East, and the rate of increase is especially pronounced in low- and middle-income countries (1, 2). Additionally, cardiovascular disease remains the principal cause of death in this population (3). Individuals with DM have a two- to four-fold increased risk of cardiovascular (CV) events (4, 5). This risk is further exacerbated in individuals who have concomitant dyslipidemia (5, 6). The atherogenic lipid profile in DM accelerates the progression of atherosclerosis and markedly increases the risk of CV adverse outcomes and mortality. Despite advances in therapy, DM still have an elevated risk for CV disease and its complications, even when treated with statins and lifestyle modifications (5).

Current clinical guidelines suggest aggressive lipid treatment in diabetes to mitigate cardiovascular risk. Statin is the first-line medication for dyslipidemia, particularly in individuals aged 40–75 years, regardless of initial lipid concentrations (4, 7, 8). The primary therapeutic goal is to lower low-density lipoprotein cholesterol, since multiple large-scale trials have demonstrated the cardiovascular benefits of statin treatment in this population. However, despite optimal statin treatment, many individuals with DM do not attain target lipid levels, and their risk of CV adverse outcomes remains high (5, 7, 8).

Proprotein convertase subtilisin/kexin type 9 inhibitors (PCSK9is) who’s use has been widely accepted in both familial homozygous and heterozygous hypercholesterolemias and their variants (9, 10), have recently been recognized as a promising adjunctive therapy for DM and dyslipidemia at high risk of CV events (8). Previous studies have shown that these inhibitors can lower low-density lipoprotein cholesterol by up to 60%, with some studies reporting even greater reductions (11). These agents have demonstrated the ability to further decrease low-density lipoprotein (LDL) cholesterol levels and mitigate the risk of major adverse CV events (MACEs) in high-risk populations (12). Importantly, PCSK9is are effective in individuals with DM and do not significantly worsen glycemic control (11, 13). However, evidence regarding their long-term effectiveness specifically in diabetic populations remains limited. Given these gaps in the literature, this real-world study was conducted to clarify the effectiveness of PCSK9i in the management of dyslipidemia among T2D patients.

## Methods

### Dataset

This study utilized TriNetX research network, that integrates data from participating healthcare organizations worldwide. The database includes longitudinal information on demographics, diagnoses, prescriptions, procedures, and selected laboratory findings. The study protocol followed the STROBE guidelines for observational research and obtained approval from the Institutional Review Board of Chi Mei Medical Center (approval number: 11402-E02).

### Study design and population

We identified adults aged with both T2D and dyslipidemia who began treatment with either a PCSK9i or a statin between January 1, 2015, and April 30, 2025. T2D and dyslipidemia were determined using the International Classification of Diseases, Tenth Revision, Clinical Modification (ICD-10-CM) codes E11 and E78, respectively. Two mutually exclusive cohorts were then established. The PCSK9i cohort included patients who initiated alirocumab (RxNorm 1659152), evolocumab (RxNorm 1665684), or inclisiran (RxNorm 2588243) after being diagnosed with both conditions. The comparison cohort consisted of individuals who received statins, identified by the Anatomical Therapeutic Chemical (ATC) classification code C10AA. The index date was defined as the earliest documented prescription date, and a 12-month look-back duration before the index date was used to assess baseline clinical characteristics.

To maintain a new-user framework and reduce misclassification, we excluded any participant with prior exposure to PCSK9is in the PCSK9i cohort. Prior statin use was allowed in this group, given the stepwise intensification of lipid-lowering therapy in clinical practice. Conversely, individuals assigned to the statin cohort were excluded if they had previously used either statins or PCSK9 inhibitors or if they later initiated PCSK9i therapy during the observation window. Patients who experienced any of the prespecified study outcomes before cohort entry or lacked follow-up information after the index date were also removed from the analysis. Comprehensive coding schemes for diagnoses, procedures, medications, and laboratory parameters used to define all variables are detailed in **Supplementary Table S1**.

Furthermore, propensity scores were generated using logistic regression models to estimate the likelihood of each participant being classified into the comparison cohort. A one-to-one nearest-neighbor matching method was applied, using a caliper width equal to 0.1 of the pooled standard deviation of the logit of the propensity score. Participants from the smaller cohort were matched to those with the closest estimated scores in the larger group. Covariate balance between the two matched groups was considered acceptable while the values of standardized mean difference (SMD) for each variable was less than 0.1 (14).

The propensity score matching (PSM) process included a broad range of baseline variables to ensure comparability between the cohorts. Demographic characteristics included age, sex, and race, and associated comorbid conditions. In addition, diabetes-related complications such as renal, ophthalmic, neurological, and circulatory manifestations were considered. Medication use at baseline was evaluated for both antihypertensive and antidiabetic therapies. Laboratory variables included body mass index, hemoglobin A1c (HbA1c), estimated glomerular filtration rate (eGFR), serum albumin less than 3.5 grams per deciliter, high-density lipoprotein cholesterol (HDL-C), low-density lipoprotein cholesterol (LDL-C), total cholesterol, and triglycerides. A full list of coding definitions and operational details for each covariate is presented in **Supplementary Table S2**.

### Outcomes and follow-up

The primary endpoint of this study was a composite outcome encompassing all-cause mortality, major adverse cardiovascular events (MACE), and major adverse kidney events (MAKE). Each component of this composite measure was also analyzed separately as a secondary outcome. MACE included clinical events such as cerebral infarction, hemorrhagic stroke, acute myocardial infarction, and cardiac arrest, whereas MAKE referred to the occurrence of end-stage kidney disease, the resumption of dialysis, or the initiation of new dialysis treatment, as outlined in **Supplementary Table S3** (15–17). Follow-up commenced on the index date and extended until the earliest occurrence among three conditions: the onset of any study outcome, death from any cause, or the completion of a five-year observation period.

### Additional analysis

Subgroup analyses were pre-specified according to demographic and clinical factors. The demographic subgroups included sex and two age categories, namely 18 to 64 years and 65 years or older. Additional subgroup comparisons were conducted according to the presence of atherosclerotic cardiovascular disease, the specific type of PCSK9i used, concurrent statin therapy among PCSK9i users, and baseline levels of LDL-C, eGFR, and HbA1c.

Two negative control outcomes including skin cancer and hernia, were evaluated to identify possible residual confounding. E values were tested to assess the effect of an unmeasured confounder that could fully explain the observed association (18). A landmark analysis was performed to examine whether the estimated effects varied across different follow up intervals (19) and additional sensitibity test was also conducted for those with concurrent use of fibrates.

### Statistical analysis

PSM was used to match demographic characteristics before performing the primary analysis, subgroup evaluations, and sensitivity assessments. All tests were conducted in the TriNetX research environment.

## Results

### Study cohort

**Figure 1:**
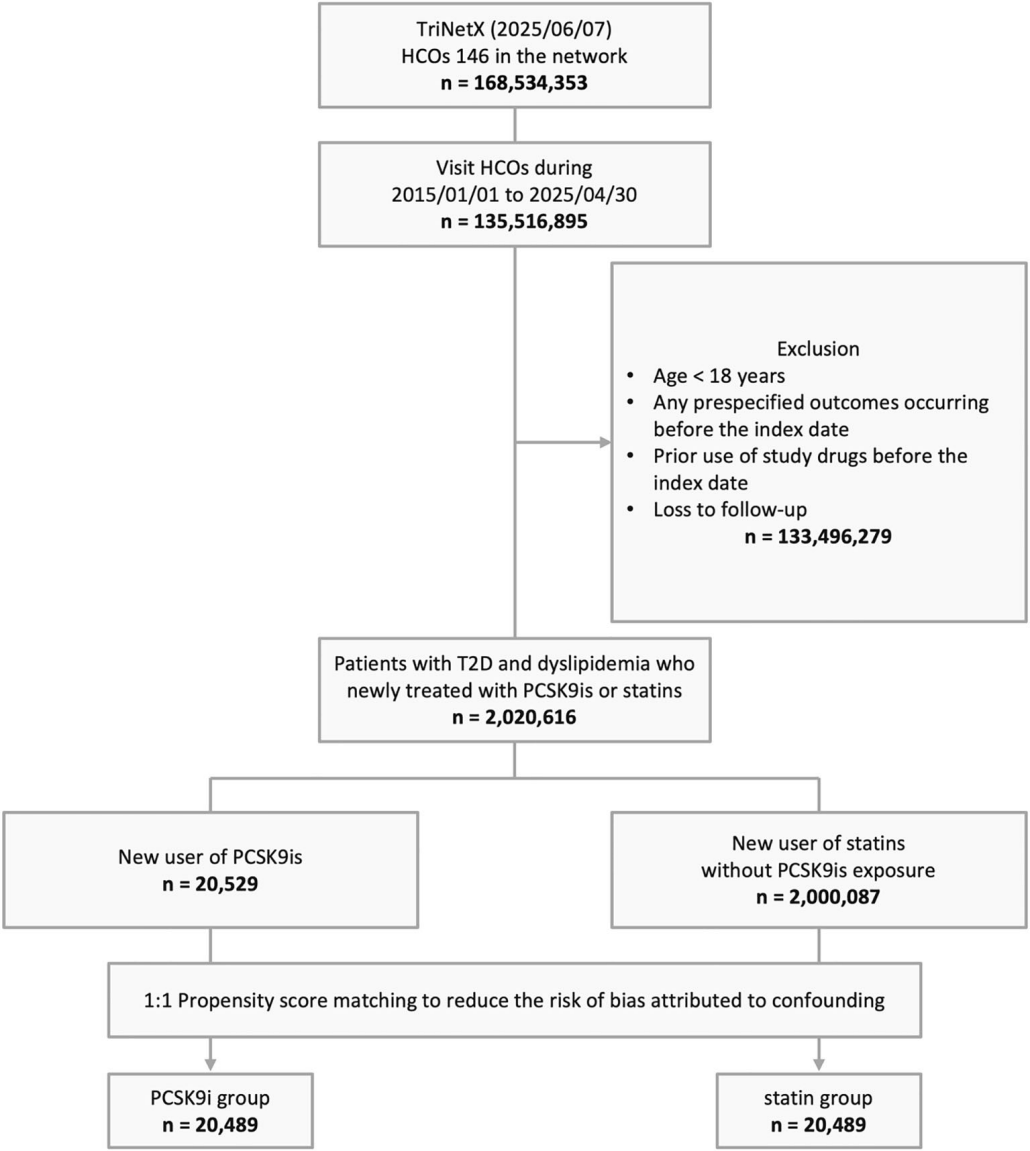
The algorithm of patient selection. HCO, healthcare organization; T2D, type 2 diabetes; PCSK9i, proprotein convertase subtilisin/kexin type 9 inhibitor.

Based on a screening of 168,534,353 individuals across 146 HCOs TriNetX networks as of June 7, 2025, a total of 135,516,895 individuals with documented HCO visits from January 1, 2015, to April 30, 2025, were identified. After applying exclusion criteria, 2,020,616 individuals with T2D and dyslipidemia who were newly treated with PCSK9i or statins were included, comprising 20,529 new users of PCSK9i and 2,000,087 new users of statins. Following PSM, 20,489 individuals were assigned to each of the PCSK9i and statin groups (**Figure 1**).

### Study subjects

Prior to match, the PCSK9i group was older than thethan those in the statin group (66.4 ± 9.8 vs. 64.5 ± 12.7 years), with notable differences in sex and race distributions (**Table 1**). The PCSK9i group had a more hypertension, overweight/obesity, chronic kidney disease, ischemia heart disease, peripheral vascular disease, cerebrovascular disease, heart failure, atrial fibrillation/flutter, atherosclerosis, chronic liver disease, chronic lower respiratory disease, and malignancy (all SMDs > 0.1). Moreover, the PCSK9i group exhibited exhibited more T2D-related complications, including kidney, ophthalmic, neurological, and circulatory involvement, and HbA1c ≥ 9%. Use of antihypertensive and anti-diabetic medications was higher in the PCSK9i group. Lastly, PCSK9i group had higher prevalence of BMI ≥ 30 kg/m^2^, albumin < 3.4 g/dL. Regarding lipid profile, PCSK9i had higher LDL-C, total cholesterol, triglyceride. After PSM, 191,273 individuals were included in each group (**Table 1**).

**Table 1:** Baseline characteristics before and after matching.

Variables	Before matching			After matching		
	PCSK9i group (n=20,529)	Statin group (n=2,000,087)	Standardized mean difference	PCSK9i group (n=20,489)	Statin group (n=20,489)	Standardized mean difference
Age at index, years						
Mean (SD)	66.4 (9.8)	64.5 (12.7)	0.166	66.3 (9.8)	66.3 (11.9)	0.009
Sex, n (%)						
Female	10,542 (51.4)	919,121 (45.9)	0.109	10,523 (51.4)	10,582 (51.6)	0.006
Male	9,151 (44.6)	1,011,801 (50.6)	0.120	9,132 (44.6)	9,032 (44.1)	0.010
Race, n (%)						
White	14,659 (71.4)	1,172,963 (58.6)	0.271	14,624 (71.4)	14,709 (71.8)	0.009
Black or African American	2,680 (13.1)	329,942 (16.5)	0.097	2,677 (13.1)	2,593 (12.7)	0.012
Asian	663 (3.2)	129,857 (6.5)	0.152	663 (3.2)	654 (3.2)	0.002
Other Race	481 (2.3)	63,825 (3.2)	0.052	481 (2.3)	472 (2.3)	0.003
Unknown Race	1,910 (9.3)	276,969 (13.8)	0.142	1,908 (9.3)	1,884 (9.2)	0.004
Comorbidities, n (%)						
Alcohol related disorders	320 (1.6)	32,817 (1.6)	0.006	318 (1.6)	293 (1.4)	0.010
Nicotine dependence	1,907 (9.3)	156,203 (7.8)	0.053	1,902 (9.3)	1,823 (8.9)	0.013
Hypertension	16,187 (78.8)	1,141,853 (57.1)	0.480	16,152 (78.8)	16,055 (78.4)	0.012
Overweight and obesity	6,886 (33.5)	406,297 (20.3)	0.302	6,857 (33.5)	6,735 (32.9)	0.013
Malnutrition	269 (1.3)	30,925 (1.5)	0.020	269 (1.3)	217 (1.1)	0.023
Chronic kidney disease	4,679 (22.8)	296,404 (14.8)	0.205	4,662 (22.8)	4,491 (21.9)	0.020
Ischemic heart diseases	11,578 (56.4)	408,532 (20.4)	0.796	11,538 (56.3)	11,315 (55.2)	0.022
Peripheral vascular diseases	2,238 (10.9)	85,695 (4.3)	0.252	2,227 (10.9)	2,257 (11)	0.005
Cerebrovascular diseases	3,471 (16.9)	171,512 (8.6)	0.252	3,459 (16.9)	3,419 (16.7)	0.005
Heart failure	3,959 (19.3)	205,394 (10.3)	0.256	3,933 (19.2)	3,787 (18.5)	0.018
Atrial fibrillation and flutter	2,868 (14)	173,488 (8.7)	0.168	2,859 (14)	2,732 (13.3)	0.018
Atherosclerosis	1,967 (9.6)	72,887 (3.6)	0.241	1,955 (9.5)	1,988 (9.7)	0.005
Chronic liver disease	2,574 (12.5)	111,187 (5.6)	0.245	2,563 (12.5)	2,560 (12.5)	< 0.001
Chronic lower respiratory diseases	4,490 (21.9)	272,066 (13.6)	0.218	4,476 (21.8)	4,441 (21.7)	0.004
Neoplasms	4,655 (22.7)	289,003 (14.4)	0.213	4,643 (22.7)	4,673 (22.8)	0.003
T2D related complications, n (%)						
Kidney complications	3,427 (19.7)	210,554 (12)	0.213	3,408 (19.7)	3,299 (19.1)	0.016
Ophthalmic complications	1,346 (7.8)	72,932 (4.2)	0.152	1,336 (7.7)	1,303 (7.5)	0.007
Neurological complications	3,194 (18.4)	182,333 (10.4)	0.23	3,172 (18.3)	3,248 (18.8)	0.011
Circulatory complications	2,050 (11.8)	74,851 (4.3)	0.28	2,031 (11.7)	2,092 (12.1)	0.011
Antihypertensives, n (%)						
ACEis	4,469 (21.8)	293,625 (14.7)	0.185	4,466 (21.8)	4,384 (21.4)	0.010
ARBs	5,994 (29.2)	218,324 (10.9)	0.469	5,962 (29.1)	6,071 (29.6)	0.012
Beta blockers	9,864 (48)	408,725 (20.4)	0.609	9,828 (48)	9,557 (46.6)	0.026
Calcium channel blockers	6,585 (32.1)	285,674 (14.3)	0.432	6,562 (32)	6,442 (31.4)	0.013
Diuretics	7,649 (37.3)	379,347 (19)	0.416	7,623 (37.2)	7,503 (36.6)	0.012
Anti-diabetic drugs, n (%)						
Biguanides	6,036 (29.4)	434,997 (21.7)	0.176	6,019 (29.4)	5,972 (29.1)	0.005
Sulfonylureas	2,241 (10.9)	177,845 (8.9)	0.068	2,235 (10.9)	2,222 (10.8)	0.002
Thiazolidinediones	470 (2.3)	30,238 (1.5)	0.057	470 (2.3)	499 (2.4)	0.009
Alpha glucosidase inhibitors	39 (0.2)	4,067 (0.2)	0.003	39 (0.2)	34 (0.2)	0.006
DPP4i	1,306 (6.4)	97,904 (4.9)	0.064	1,305 (6.4)	1,275 (6.2)	0.006
SGLT2i	3,453 (16.8)	67,282 (3.4)	0.458	3,415 (16.7)	3,466 (16.9)	0.007
GLP1RA	4,040 (19.7)	86,588 (4.3)	0.486	4,010 (19.6)	4,117 (20.1)	0.013
Insulin	7,706 (37.5)	408,609 (20.4)	0.384	7,672 (37.4)	7,511 (36.7)	0.016
Body mass index, kg/m^2^						
≥ 30, n (%)	9,978 (48.6)	679,762 (34)	0.301	9,952 (48.6)	9,984 (48.7)	0.003
Hemoglobin A1c, %						
≥ 9, n (%)	2,239 (10.9)	213,931 (10.7)	0.007	2,234 (10.9)	2,117 (10.3)	0.019
eGFR, n(%), mL/min/1.73m^2^						
< 15, n (%)	659 (3.2)	75,911 (3.8)	0.032	658 (3.2)	582 (2.8)	0.022
Albumin, g/dL						
< 3.5, n (%)	3,427 (16.7)	247,643 (12.4)	0.123	3,417 (16.7)	3,327 (16.2)	0.012
Cholesterol in HDL, mg/dL						
Mean (SD)	43.5 (16.5)	43.6 (16.8)	0.006	43.5 (16.5)	43.7 (16.7)	0.009
< 40, n (%)	6,486 (31.6)	350,465 (17.5)	0.332	6,462 (31.5)	6,340 (30.9)	0.013
Cholesterol in LDL, mg/dL						
Mean (SD)	128.6 (53.4)	99.9 (42.5)	0.593	128.5 (53.5)	116.7 (43.8)	0.242
≥ 100, n (%)	11,044 (53.8)	412,574 (20.6)	0.731	11,005 (53.7)	11,010 (53.7)	< 0.001
Cholesterol, mg/dL						
Mean (SD)	212.1 (63.7)	178.8 (54.7)	0.561	212.1 (63.8)	198.6 (53.7)	0.229
≥ 200, n (%)	9,117 (44.4)	294,530 (14.7)	0.688	9,081 (44.3)	9,066 (44.2)	0.001
Triglyceride, mg/dL						
Mean (SD)	209.2 (215.8)	184.8 (205.1)	0.116	209.2 (215.9)	199.3 (180.9)	0.049
≥ 150, n (%)	9,621 (46.9)	431,436 (21.6)	0.553	9,587 (46.8)	9,598 (46.8)	0.001

ACEi, angiotensin-converting enzyme inhibitor; ARB, angiotensin receptor blocker; eGFR, estimated Glomerular filtration rate; DPP4i, dipeptidyl peptidase 4 inhibitor; GLP1RA, glucagon-like peptide-1 receptor agonist; HDL, high-density lipoprotein; LDL, low-density lipoprotein; PCSK9i, proprotein convertase subtilisin/kexin type 9 inhibitor; SD, standard deviation; SGLT2i, sodium-glucose cotransporter 2 inhibitor; T2D, type 2 diabetes.

### Primary outcome

**Figure 2:**
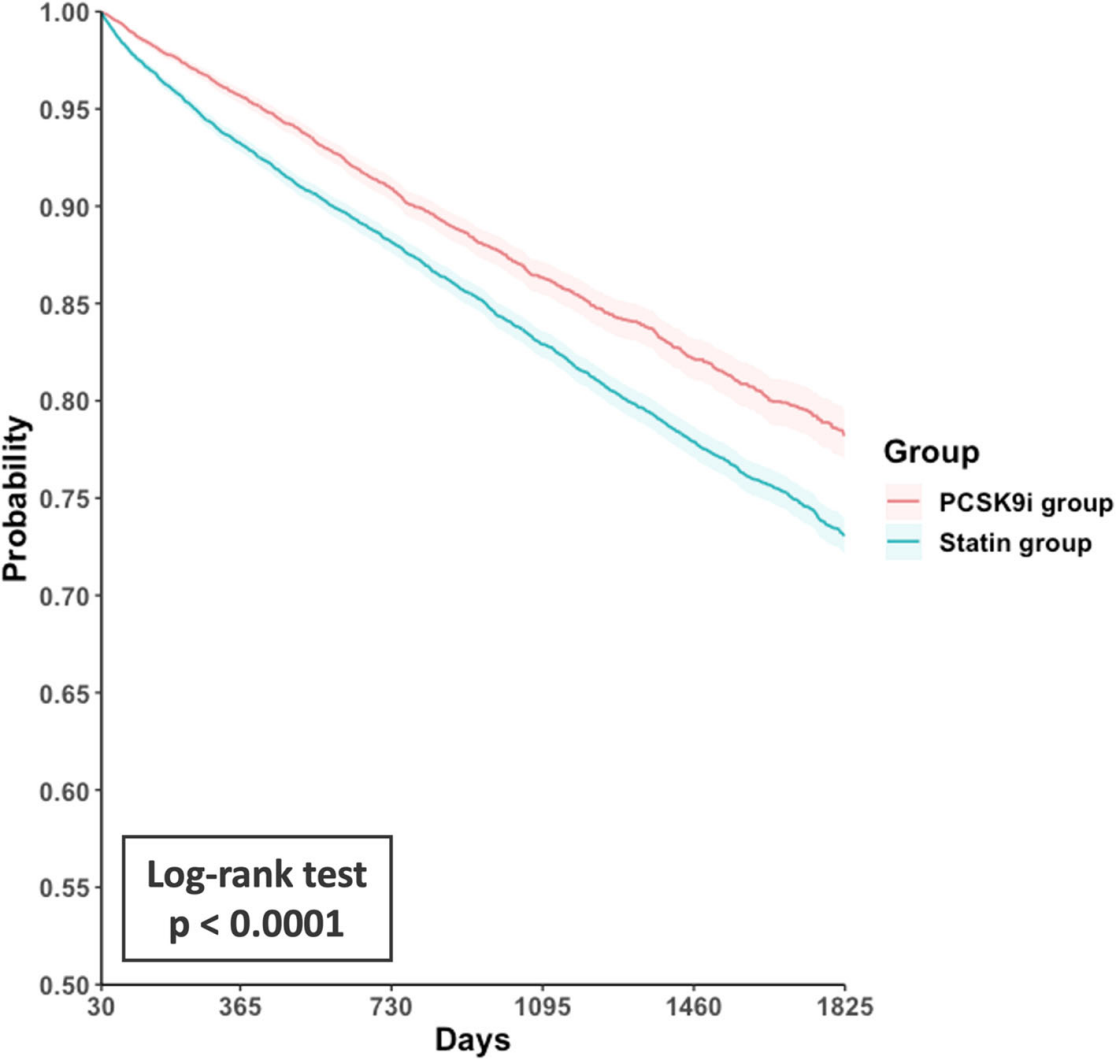
Kaplan-Meier curves for event-free survival from the primary composite outcome in the PCSK9i group versus the statin group. PCSK9i, proprotein convertase subtilisin/kexin type 9 inhibitor.

Compared with the statin group, the PCSK9i group exhibited a lower incidence of primary outcomes (hazard ratio [HR], 0.75; 95% confidence interval [CI], 0.70–0.81; **Table 2**). The corresponding E-value was 2.0. Survival analysis revealed a high accumulated incidence of free-from primary outcomes in the PCSK9i group (log-rank test, p < 0.0001, **Figure 2**).

**Table 2:** Hazard ratios of primary and secondary outcomes for the comparison of the PCSK9i group and the statin group.

Outcomes	PCSK9i group (n=20,489)		Statin group (n=20,489)		HR (95% CI)	E-value (LCL)
	Event	Incidence rate (%)	Event	Incidence rate (%)		
Primary outcome						
Composite outcome	1,209	5.9	2,652	12.9	0.75 (0.70,0.81)	2.0 (1.8)
Secondary outcomes						
All-cause mortality	957	4.7	2,314	11.3	0.65 (0.60,0.70)	2.5 (2.2)
MACE	866	4.2	1,735	8.5	0.83 (0.76,0.90)	1.7 (1.5)
MAKE	277	1.4	605	3.0	0.70 (0.61,0.81)	2.2 (1.8)

CI, confidence interval; HR, hazard ratio; MACE, major adverse cardiovascular event; LCL, lower confidence limit; MAKE, major adverse kidney event; PCSK9i, proprotein convertase subtilisin/kexin type 9 inhibitor.

**Figure 3:**
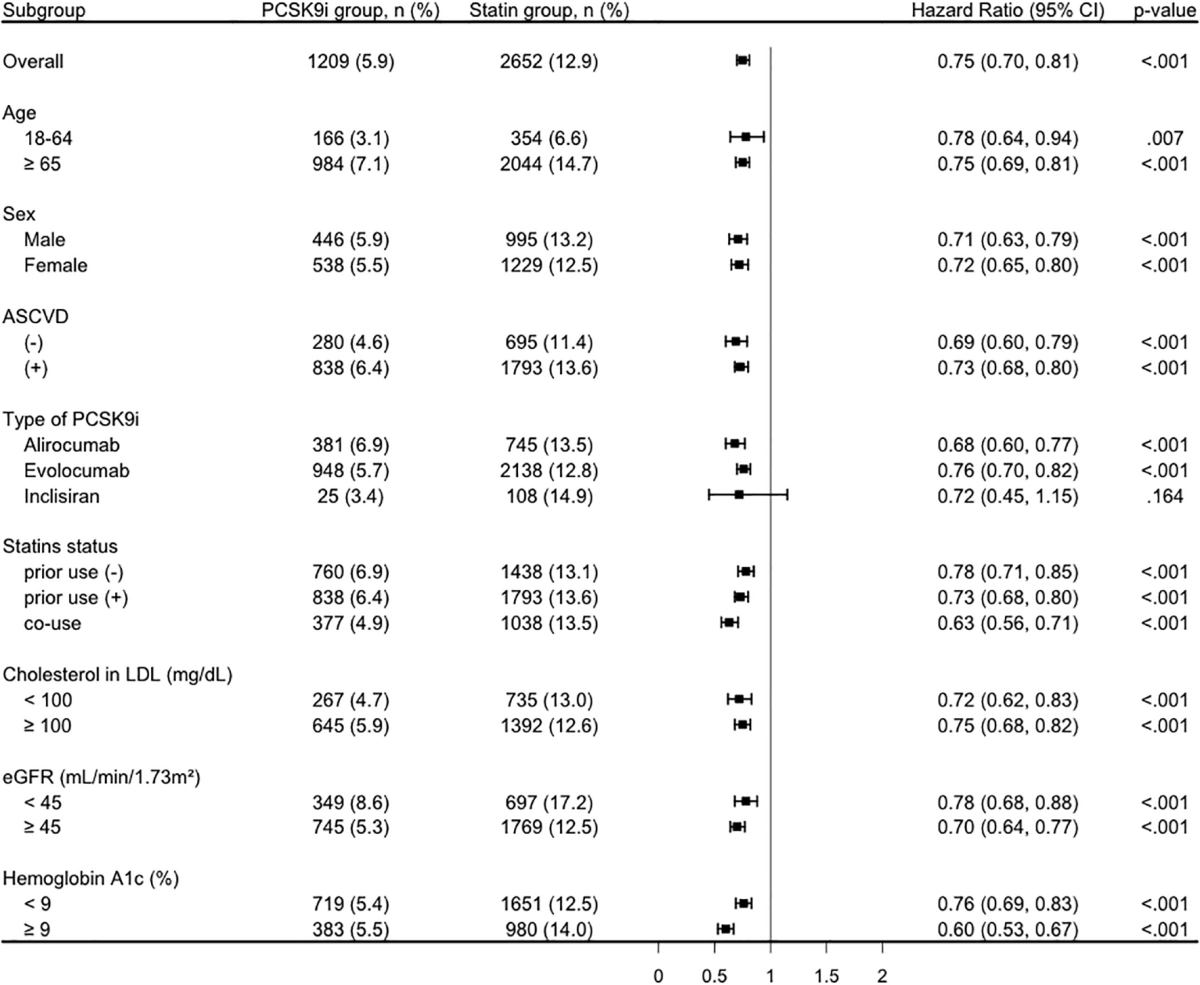
Subgroup analysis of the primary composite outcome comparing the PCSK9i group and the statin group. ASCVD, atherosclerotic cardiovascular disease; CI, confidence interval; eGFR, estimated Glomerular filtration rate; LDL, low-density lipoprotein; PCSK9i, proprotein convertase subtilisin/kexin type 9 inhibitor.

The reduced risk associated with PCSK9i (HR < 1) was consistent across all stratified analyses (**Figure 3**). In detail, HR was 0.78 (95% CI, 0.64-0.94) among patients aged 18–64 years and 0.75 (95% CI, 0.69-0.81) among those aged ≥65 years. In both male and female patients, the association remained significant, with HRs of 0.71 (95% CI, 0.63-0.79) and 0.72 (95% CI, 0.65-0.80), respectively. Similarly, patients with or without a history of ASCVD demonstrated a reduced risk (HR, 0.73 [95% CI, 0.68-0.80] and 0.69 [95% CI, 0.60-0.79], respectively). Among the different PCSK9 inhibitors, alirocumab (HR, 0.68; 95% CI, 0.60-0.77) and evolocumab (HR, 0.76; 95% CI, 0.70-0.82) showed significant associations, while inclisiran did not (HR, 0.72; 95% CI, 0.45-1.15). Notably, the benefit persisted regardless of prior statin use or PCSK9i-statin co-use, with HRs ranging from 0.63 to 0.78. The favorable association was also consistent across subgroups with different LDL levels, kidney function (eGFR), and HbA1c level.

### Secondary outcomes

The PCSK9i group exhibited consistently lower risks of all-cause mortality (HR, 0.65; 95% CI, 0.60-0.70), MACE (HR, 0.83; 95% CI, 0.76-0.90), and MAKE (HR, 0.70; 95% CI, 0.61-0.81) (**Table 2**). The corresponding E-values were 2.5, 1.7, and 2.2 for the analyses of all-cause mortality, MACE, and MAKE, respectively.

### Additional tests

In landmark analyses, the association between PCSK9i use and risk of composite outcome remained robust across different time windows, with HRs ranging from 0.73 to 0.80, all statistically significant (P < 0.0001) (**Supplementary Table S4**). Additionally, a similar trend was observed among those concurrently using fibrates (**Supplementary Table S5**). Lastly, no significant associations were observed between PCSK9i use and negative control outcomes, including skin cancer (HR 0.93, 95% CI 0.80–1.08) and hernia (HR 1.01, 95% CI 0.93–1.10) (**Supplementary Table S6**), supporting the validity of the findings.

## Discussion

In this large cohort study, PCSK9i use was associate with significantly lower incidences of clinical outcomes, including all-cause mortality, MACE, and MAKE, compared to statin therapy in individuals with T2D and dyslipidemia. The primary outcome showed a 25% risk reduction (HR, 0.75), with robustness confirmed by an E-value of 2.0. Benefits were consistent across key subgroups defined by age, sex, ASCVD history, LDL-C, eGFR, and HbA1c, and were observed with both alirocumab and evolocumab. Secondary outcomes showed similarly favorable associations, and all the findings were supported by sensitivity and landmark analyses. These findings hold significant clinical relevance, as patients with T2D continue to face persistent residual cardiovascular and renal risk despite guideline-directed statin therapy. The consistent benefit observed across diverse patient subgroups further supports the broad clinical utility of PCSK9i in managing high-risk patients with T2D and dyslipidemia.

Our findings align with previous randomized trials (20, 21) demonstrating cardiovascular risk reduction with PCSK9is in high-risk populations, but expand the evidence by focusing specifically on patients with T2D in routine clinical practice. However, these trials (20, 21) included relatively selected populations and did not focus exclusively on patients with T2D, a group known to have persistent residual cardiovascular and renal risk despite statin use. Our study contributes to the expanding real-world evidence by showing that PCSK9i therapy provides consistent clinical benefits in the population of individuals with T2D and dyslipidemia, reflecting everyday clinical practice and diverse patient profiles (22).

The clinical benefits of PCSK9is can be explained by several interrelated mechanisms. PCSK9 inhibitors act by binding to the PCSK9 protein, thereby preventing it from promoting the degradation of LDL receptors on hepatocytes. This mechanism enhances LDL receptor recycling and increases receptor density, which futher facilitates greater removal of LDL cholesterol from the circulation and results in substantial reductions in LDL-C levels (11, 13). Beyond LDL-C lowering, PCSK9 inhibition may also reduce levels of lipoprotein(a) by approximately 20–30%, which could contribute to further cardiovascular risk reduction (23). Additionally, PCSK9is may exert pleiotropic effects such as improving endothelial function (24–26) and reducing inflammation (27, 28), which are important in the pathophysiology of atherosclerosis and diabetic vascular complications. The combined lipid-lowering and potential vascular protective effects likely underlie the observed reductions in adverse clinical outcomes in patients with T2D and dyslipidemia treated with PCSK9is.

Strengths of this study include its real-world design using a global electronic health record (EHR) network, which enhances generalizability to routine clinical practice. The substantial sample size and extended follow-up duration allowed for robust estimation of treatment effects in diverse patient subgroups. PSM was applied to balance a wide range of baseline characteristics between treatment groups, reducing the impact of measured confounding. Multiple sensitivity analyses and stratified analyses confirmed the consistency of findings. Furthermore, the application of E-values provided evidence that the observed associations are likely resilient to the influence of unmeasured confounding.

However, there were some limitations. As with other observational researches based on EHR data, the potential for residual confounding remains, although the high E-values were observed in most of analyses. Misclassification of exposures or outcomes is possible due to reliance on structured coding and lack of manual validation. The non-randomized design inherently limits causal inference. In particular, data on the duration and severity of T2D were not available, which may influence clinical outcomes. However, we attempted to mitigate this limitation by matching the two groups on multiple indicators of diabetes burden, including the prevalence of diabetes-related complications, HbA1c levels, and the use of various anti-diabetic medications. Finally, the subgroup analysis of inclisiran was limited by a small sample size, precluding definitive conclusions about its comparative effectiveness relative to alirocumab and evolocumab.

In conclusion, this large real-world study based on a global EHR network demonstrates that treatment with PCSK9i is associated with significantly reduced risks of adverse clinical outcomes in individuals with T2D and dyslipidemia, compared with statin therapy. Our findings suggest the consideration of PCSK9i as an important component of intensified cholesterol management in individuals with T2D. By providing real-world evidence from a large and diverse population, this study contributes to the growing support for broader integration of PCSK9i into contemporary cardiovascular and renal risk management in T2D with dyslipidemia.

## Data Availability

The original contributions presented in the study are included in the article/**Supplementary Material**. Further inquiries can be directed to the corresponding author.
