# Supplementary material for: Effectiveness of PCSK9 inhibitors versus statins in type 2 diabetes and dyslipidemia: a propensity-matched study
Source: Front Endocrinol (Lausanne). 2025 Nov 19;16:1709009. doi: 10.3389/fendo.2025.1709009 (PMC12672303; doi:10.3389/fendo.2025.1709009)
Supplement: Supplementary file 1 [file Table1.docx]

**Table S1.** Demographic, diagnostic, procedural, medication, visit, and laboratory codes utilized in the definition of the cohorts.

| **Category** | **Code** | **Description** |
| --- | --- | --- |
| **PCSK9i group** | | |
| **#1**: At least 18 years old | | |
| Demographics | Age | Age (at least 18 years) |
| **#2**: Patients with type 2 diabetes and dyslipidemia treated with PCSK9i between Jul 1, 2015 and Apr 30, 2025  (# 2.1 must be fulfilled within 1 year after #2.2) | | |
| **#2.1**: Patients with PCSK9i | | |
| Medication | NLM:RXNORM:1659152 | alirocumab |
| Medication | NLM:RXNORM:1665684 | evolocumab |
| Medication | NLM:RXNORM:2588243 | inclisiran |
| **#2.2**: Patients with type 2 diabetes mellitus and dyslipidemia | | |
| Diagnosis | UMLS:ICD10CM:E11 | Type 2 diabetes mellitus |
| Diagnosis | UMLS:ICD10CM:E78 | Disorders of lipoprotein metabolism and other lipidemias |
| #3: **#3:** Without prior outcomes (cannot have any of the following)  #3 must be fulfilled before #2.1 | | |
| Diagnosis | UMLS:ICD10CM:I21 | Acute myocardial infarction |
| Diagnosis | UMLS:ICD10CM:I22 | Subsequent ST elevation (STEMI) and non-ST elevation (NSTEMI) myocardial infarction |
| Diagnosis | UMLS:ICD10CM:I61 | Nontraumatic intracerebral hemorrhage |
| Diagnosis | UMLS:ICD10CM:I62 | Other and unspecified nontraumatic intracranial hemorrhage |
| Diagnosis | UMLS:ICD10CM:I63 | Cerebral infarction |
| Diagnosis | UMLS:ICD10CM:I46.2 | Cardiac arrest due to underlying cardiac condition |
| Diagnosis | UMLS:ICD10CM:I46.9 | Cardiac arrest, cause unspecified |
| Diagnosis | UMLS:ICD10CM:N18.6 | End stage renal disease |
| Diagnosis | UMLS:ICD10CM:Z99.2 | Dependence on renal dialysis |
| Procedure | UMLS:CPT:1012740 | Dialysis Services and Procedures |
| Procedure | UMLS:CPT:1029674 | Dialysis Circuit Procedures |
| **#4**: Incident user (cannot have the following)  #4 must be fulfilled before #2.1 | | |
| Medication | NLM:RXNORM:1659152 | alirocumab |
| Medication | NLM:RXNORM:1665684 | evolocumab |
| Medication | NLM:RXNORM:2588243 | inclisiran |
| **#5:** Have follow-up records up to five years after the index date (have any of the following) | | |
| visit | TNX:Visit | Visit |
| demographics | Deceased | Deceased |
| diagnosis | UMLS:ICD10CM:R99 | Ill-defined and unknown cause of mortality |
| **Statins group** | | |
| **#1**: At least 18 years old | | |
| Demographics | Age | Age (at least 18 years) |
| **#2**: Patients with type 2 diabetes and dyslipidemia treated with statins between Jul 1, 2015 and Apr 30, 2025  (# 2.1 must be fulfilled within 1 year after #2.2) | | |
| **#2.1**: Patients with statins | | |
| Medication | NLM:ATC:C10AA | HMG CoA reductase inhibitors |
| **#2.2**: Patients with type 2 diabetes mellitus and dyslipidemia | | |
| Diagnosis | UMLS:ICD10CM:E11 | Type 2 diabetes mellitus |
| Diagnosis | UMLS:ICD10CM:E78 | Disorders of lipoprotein metabolism and other lipidemias |
| #3: **#3:** Without prior outcomes (cannot have any of the following)  #3 must be fulfilled before #2.1 | | |
| Diagnosis | UMLS:ICD10CM:I21 | Acute myocardial infarction |
| Diagnosis | UMLS:ICD10CM:I22 | Subsequent ST elevation (STEMI) and non-ST elevation (NSTEMI) myocardial infarction |
| Diagnosis | UMLS:ICD10CM:I61 | Nontraumatic intracerebral hemorrhage |
| Diagnosis | UMLS:ICD10CM:I62 | Other and unspecified nontraumatic intracranial hemorrhage |
| Diagnosis | UMLS:ICD10CM:I63 | Cerebral infarction |
| Diagnosis | UMLS:ICD10CM:I46.2 | Cardiac arrest due to underlying cardiac condition |
| Diagnosis | UMLS:ICD10CM:I46.9 | Cardiac arrest, cause unspecified |
| Diagnosis | UMLS:ICD10CM:N18.6 | End stage renal disease |
| Diagnosis | UMLS:ICD10CM:Z99.2 | Dependence on renal dialysis |
| Procedure | UMLS:CPT:1012740 | Dialysis Services and Procedures |
| Procedure | UMLS:CPT:1029674 | Dialysis Circuit Procedures |
| **#4**: Incident user (cannot have the following)  #4 must be fulfilled before #2.1 | | |
| Medication | NLM:ATC:C10AA | HMG CoA reductase inhibitors |
| **#5**: Exclude PCSK9i (cannot have any of the following)  #6 must be fulfilled within 5 years after #2.1 | | |
| Medication | NLM:RXNORM:1659152 | alirocumab |
| Medication | NLM:RXNORM:1665684 | evolocumab |
| Medication | NLM:RXNORM:2588243 | inclisiran |
| **#6:** Have follow-up records up to five years after the index date (have any of the following) | | |
| visit | TNX:Visit | Visit |
| demographics | Deceased | Deceased |
| diagnosis | UMLS:ICD10CM:R99 | Ill-defined and unknown cause of mortality |

ICD10CM, International Classification of Diseases, Tenth Revision, Clinical Modification

NLM, National Library of Medicine

RXNORM, medical prescription normalized

UMLS, Unified Medical Language System

**Table S2.** Definitions of covariates coding used in this study.

| **Code** | **Description** |
| --- | --- |
| AI | Age at Index |
| 2106-3 | White |
| UNK | Unknown Race |
| F | Female |
| 2054-5 | Black or African American |
| M | Male |
| 2131-1 | Other Race |
| 2028-9 | Asian |
| F17 | Nicotine dependence |
| F10 | Alcohol related disorders |
| E40-E46 | Malnutrition |
| E66 | Overweight and obesity |
| I10 | Essential (primary) hypertension |
| I20-I25 | Ischemic heart diseases |
| I48 | Atrial fibrillation and flutter |
| I60-I69 | Cerebrovascular diseases |
| J40-J4A | Chronic lower respiratory diseases |
| K70-K77 | Diseases of liver |
| C00-D49 | Neoplasms |
| N18 | Chronic kidney disease (CKD) |
| I70 | Atherosclerosis |
| I73 | Other peripheral vascular diseases |
| I63 | Cerebral infarction |
| I50 | Heart failure |
| E11.2 | Type 2 diabetes mellitus with kidney complications |
| E11.3 | Type 2 diabetes mellitus with ophthalmic complications |
| E11.4 | Type 2 diabetes mellitus with neurological complications |
| E11.5 | Type 2 diabetes mellitus with circulatory complications |
| CV100 | BETA BLOCKERS/RELATED |
| CV200 | CALCIUM CHANNEL BLOCKERS |
| CV700 | DIURETICS |
| CV800 | ACE INHIBITORS |
| CV805 | ANGIOTENSIN II INHIBITOR |
| A10A | INSULINS AND ANALOGUES |
| A10BA | Biguanides |
| A10BB | Sulfonylureas |
| A10BF | Alpha glucosidase inhibitors |
| A10BG | Thiazolidinediones |
| A10BH | Dipeptidyl peptidase 4 (DPP-4) inhibitors |
| A10BJ | Glucagon-like peptide-1 (GLP-1) analogues |
| A10BK | Sodium-glucose co-transporter 2 (SGLT2) inhibitors |
| 39156-5 | Body Mass Index |
| 9045 | Albumin [Mass/volume] in Serum, Plasma or Blood |
| 9037 | Hemoglobin A1c/Hemoglobin.total in Blood |
| 8001 | Glomerular filtration rate/1.73 sq M.predicted [Volume Rate/Area] in Serum, Plasma or Blood by Creatinine-based formula (MDRD) |
| 9002 | Cholesterol in LDL [Mass/volume] in Serum or Plasma |
| 9001 | Cholesterol in HDL [Mass/volume] in Serum or Plasma |
| 9000 | Cholesterol [Mass/volume] in Serum or Plasma |
| 9004 | Triglyceride [Mass/volume] in Serum, Plasma or Blood |

**Table S3.** Definitions of outcomes coding used in this study.

| **Code** | **Description** |
| --- | --- |
| **1#: Composite outcomes (composite of 2# to 4#)** | |
| **2#: MACE** | |
| UMLS:ICD10CM:I21 | Acute myocardial infarction |
| UMLS:ICD10CM:I22 | Subsequent ST elevation (STEMI) and non-ST elevation (NSTEMI) myocardial infarction |
| UMLS:ICD10CM:I61 | Nontraumatic intracerebral hemorrhage |
| UMLS:ICD10CM:I62 | Other and unspecified nontraumatic intracranial hemorrhage |
| UMLS:ICD10CM:I63 | Cerebral infarction |
| UMLS:ICD10CM:I46.2 | Cardiac arrest due to underlying cardiac condition |
| UMLS:ICD10CM:I46.9 | Cardiac arrest, cause unspecified |
| **3#: MAKE** | |
| UMLS:ICD10CM:N18.6 | End stage renal disease |
| UMLS:ICD10CM:Z99.2 | Dependence on renal dialysis |
| UMLS:CPT:1012740 | Dialysis Services and Procedures |
| UMLS:CPT:1029674 | Dialysis Circuit Procedures |
| **4#: All-cause mortality** | |
| Deceased | Deceased |
| UMLS:ICD10CM:R99 | Ill-defined and unknown cause of mortality |

ICD10CM, International Classification of Diseases, Tenth Revision, Clinical Modification

NLM, National Library of Medicine

RXNORM, medical prescription normalized

UMLS, Unified Medical Language System

**Table S4.** Sensitivity analysis for risk of primary outcome using landmark analysis.

| Sensitivity analysis | PCSK9i group (n/N) | Statin group (n/N) | Hazard ratio (95% CI) | P value |
| --- | --- | --- | --- | --- |
| 3-months to 5-years | 1,007/18,575 | 2,244/18,575 | 0.76 (0.70 – 0.82) | < 0.0001 |
| 6-months to 5-years | 878/18,555 | 2,063/18,555 | 0.75 (0.69 – 0.81) | < 0.0001 |
| 1-year to 5-years | 710/18,960 | 1,711/18,960 | 0.80 (0.74 – 0.88) | < 0.0001 |
| 3-years to 5-years | 197/18,960 | 691/18,960 | 0.73 (0.62 – 0.85) | < 0.0001 |

**Table S5.** Hazard ratios for outcomes between the PCSK9i group and the statin group under concurrent fibrates use.

| Outcomes | PCSK9i group (n/N) | Statin group (n/N) | Hazard ratio (95% CI) | P value |
| --- | --- | --- | --- | --- |
| Composite outcome | 94/1474 | 191/1474 | 0.76 (0.59 – 0.98) | 0.0307 |
| All-cause mortality | 102/1474 | 174/1474 | 0.80 (0.63 – 1.03) | 0.0798 |
| MACE | 68/1474 | 126/1474 | 0.84 (0.63 – 1.13) | 0.2546 |
| MAKE | 23/1474 | 48/1474 | 0.68 (0.41 – 1.12) | 0.1288 |

**Table S6.** Hazard ratios for negative outcomes between the PCSK9i group and the statin group.

| Outcomes | Hazard ratio (95% CI) | P value |
| --- | --- | --- |
| Skin cancer | 0.93 (0.80,1.08) | 0.341 |
| Hernia | 1.01 (0.93,1.10) | 0.750 |
